# 氟马替尼联合诱导化疗并序贯异基因造血干细胞移植治疗新诊断Ph^+^急性淋巴细胞白血病6例临床观察

**DOI:** 10.3760/cma.j.issn.0253-2727.2023.02.017

**Published:** 2023-02

**Authors:** 霞英 连, 海萍 戴, 庆亚 崔, 晓文 唐

**Affiliations:** 苏州大学附属第一医院，苏州 215006 The First Affiliated Hospital of Soochow University, Suzhou 215006, China

费城染色体阳性急性淋巴细胞白血病（Ph^+^ ALL）在成人急性淋巴细胞白血病（ALL）中占20％～30％，在儿童ALL中占2％～5％[1]。美国国家综合癌症网络（NCCN）2021版指南将Ph^+^ ALL归入预后不良组。既往研究显示，Ph^+^ ALL单纯化疗的完全缓解（CR）率为60％～90％[2]，5年总生存（OS）率仅为8％～12％[3]。酪氨酸激酶抑制剂（TKI）通过竞争性结合酪氨酸激酶上腺苷三磷酸激酶（ATP）的结合位点，阻止酪氨酸激酶磷酸化，抑制携带BCR-ABL1融合基因的白血病细胞增殖，且不影响正常细胞功能[4]。Bassan等[5]应用一代TKI伊马替尼联合化疗治疗Ph^+^ ALL患者，CR率为92％，完全分子学反应（CMR）率为40％，5年OS率为38％。Ravandi等[6]采用二代TKI达沙替尼联合Hyper-CVAD方案治疗Ph^+^ ALL患者，CR率为96％，CMR率为65％，5年OS率为46％。由于TKI价格昂贵长期用药经济负担较重且有合并耐药突变的风险，相关指南仍推荐有条件的Ph^+^ ALL患者在获得缓解后行异基因造血干细胞移植（allo-HSCT）[3]。日本的一项研究显示，一代TKI伊马替尼治疗Ph^+^ ALL获得第一次CR后序贯allo-HSCT，3年OS率、无白血病存活（LFS）率、复发率分别为65％、58％、15％，均优于未应用TKI的历史对照组[7]。我们自2020年起尝试采用我国自主研发的二代TKI氟马替尼联合诱导化疗并序贯allo-HSCT治疗6例新诊断Ph^+^ ALL，获得良好疗效。

## 病例与方法

一、病例

本研究纳入2020年4月至2021年6月于苏州大学附属第一医院一线接受氟马替尼联合诱导化疗后序贯allo-HSCT的6例新诊断Ph^+^ ALL患者，对其临床资料进行回顾性分析。

二、诊断依据

诊断标准参照《中国成人急性淋巴细胞白血病诊断与治疗指南（2016版）》，结合骨髓细胞形态学、免疫表型分析、细胞遗传学、分子生物学进行诊断分型，排除慢性髓性白血病（CML）急淋变患者。采用FACS Calibur型流式细胞仪（美国Becton Dickinson公司产品）进行免疫表型分析，检测白血病细胞表面抗原表达水平。免疫表型分析提示B细胞ALL的患者，以荧光原位杂交技术（FISH）以快速辅助诊断。采用R显带技术进行染色体核型分析，按《人类细胞遗传学国际命名体制（ISCN）（2016）》描述核型异常。染色体核型分析正常或因分裂相少无法确定核型的患者，采用RT-PCR技术检测BCR-ABL1融合基因剪切型。采用RQ-PCR方法确定BCR-ABL1融合基因拷贝数，通过Sanger测序法检测ABL激酶区突变[8]。采用高通量测序靶向测序检测基因突变，用探针捕获建库方法Nextseq550系统进行脱氧核糖核酸测序，平均测序深度>1000×。

三、治疗方案

1. 化疗方案：诱导化疗均为IVP方案（去甲氧柔红霉素+长春地辛+地塞米松）。巩固治疗方案：4例患者行一个疗程中剂量阿糖胞苷+培门冬酶（MD-Ara-C+PEG-ASP），2例患者行Hyper-CVAD/MA（A/B）（环磷酰胺+长春新碱+吡柔比星+地塞米松/甲氨蝶呤+阿糖胞苷）巩固治疗。治疗期间定期采用甲氨蝶呤（MTX）、阿糖胞苷、地塞米松三联鞘内注射预防中枢神经系统白血病（CNSL）。

2. 氟马替尼用药方案：FISH或RT-PCR法检出BCR-ABL1融合基因后即加用氟马替尼，起始剂量600 mg/d，根据患者血象进行剂量调整。巩固治疗期间及化疗间歇期持续口服氟马替尼。allo-HSCT后3个月无活动性移植物抗宿主病（GVHD）的患者开始加用氟马替尼进行维持治疗。

3. 移植方案：预处理均采用改良BuCy（白消安+环磷酰胺）方案[9]。同胞全相合造血干细胞移植1例，单倍体造血干细胞移植5例。移植物均为外周血造血干细胞，中位单个核细胞输注量为8.76（5.00～13.00）×10^8^/kg，中位CD34^+^细胞输注量为2.99（2.00～6.00）×10^6^/kg。同胞全相合造血干细胞移植患者予环孢素A（CsA）联合短程MTX预防GVHD，单倍体造血干细胞移植采用抗胸腺细胞球蛋白（ATG）、CsA、霉酚酸酯（MMF）联合短程MTX预防GVHD。粒细胞及血小板植活标准参照文献[9]。以短串联重复序列（STR）评估供者造血干细胞嵌合度。

四、安全性评估

依据WHO制定的不良反应评价标准将患者不良反应级别分为0～Ⅳ度，观察氟马替尼的主要不良反应，包括水肿、四肢疼痛、皮疹、腹泻、肝肾功能及心电图情况等。

五、疗效评价及随访

形态学CR与复发的定义参照文献[10]。MMR定义为BCR-ABL1^IS^≤0.1％（ABL转录本>10 000）。完全分子学反应（CMR）指在可扩增ABL转录本水平下无法检测到BCR-ABL1转录本。无进展生存（PFS）时间指患者氟马替尼治疗开始到任何原因引起的治疗失败（包括复发、BCR-ABL1水平升高、死亡或疾病进展等）的时间；OS时间指患者氟马替尼治疗开始至因任何原因死亡或末次随访的时间。随访资料来自住院/门诊病历及电话随访。随访截止日期为2022年2月4日。

## 结果

一、临床特征

6例新诊断Ph^+^ ALL患者中男4例、女2例，中位年龄30.5（12～43）岁。初诊时中位WBC 22.46（1.88～267.05）×10^9^/L，中位PLT 56（28～127）×10^9^/L。4例患者检测到t（9;22）（q34;q11），1例为正常染色体核型，1例染色体核型分析失败。6例患者RT-PCR均检测到BCR-ABL1融合基因，且均为e1a2（P190）型。氟马替尼治疗前ABL激酶区突变位均为阴性。通过二代测序在6例患者中共检出11种伴随基因突变，分别为IKZF1、NOTCH1、BCOR、ATG2B、SMC2、BCL2、EP300、SETD2、ZRSR2、IGLL5、RUNX1。详见表1。

**表1:** 6例新诊断Ph^+^急性淋巴细胞白血病患者临床资料

例号	性别	年龄	初诊WBC（×10^9^/L）	初诊PLT（×10^9^/L）	初诊骨髓原幼细胞（％）	染色体核型	BCR-ABL1基因类型	BCR-ABL1/ABL值(％)	基因突变	ABL激酶区突变点
1	女	12	267.05	48	91	未见分裂象	P190	86.13	−	−
2	男	24	64.09	28	87	正常	P190	114.75	IKZF1、NOTCH1、BCOR、ATG2B、SMC2	−
3	男	32	22.46	35	97	45,XY,-7,t(9;22)(q34;q11),der(16)[5]/46,XY[5]	P190	109.18	BCL2、EP300、SETD2	−
4	男	29	22.46	88	86	46,XY,t(9;22)(q34;q11)[6]/46,XY[3]	P190	182.57	SETD2	
5	男	41	1.88	64	95	46,XY,t(9;22)(q34;q11)[3]/46,idem,der(15)[1]/46,XY[16]	P190	105.73	ZRSR2、IGLL5	−
6	女	43	7.64	127	66	46,XX,?i(7)(p10),t(9;22)(q34;q11)[8]/46,XX[2]	P190	100.79	RUNX1	−

二、治疗情况与临床疗效

新诊断6例Ph^+^ ALL患者均在诱导治疗开始1周内启动氟马替尼治疗。治疗28 d时均获得形态学CR，5例患者流式细胞术检测微小残留病（MRD）阴性，1例MRD阳性（治疗第38 d转阴）；1例患者获得CMR，2例患者获得MMR，3例未达到MMR（分别在治疗第78 d、59 d及50 d达MMR），中位获得MMR的时间为34 d。治疗3个月时，6例患者均获得MMR，其中3例获得CMR。allo-HSCT前处于MMR的3例患者，在allo-HSCT后1个月内均获CMR（表2）。例1在移植后4个月开始氟马替尼治疗，治疗18 d时出现BCR-ABL1融合基因转阳，并且检测到ABL激酶区T315I突变。予更换TKI，并先后予利妥昔单抗、CD19/22 CAR-T细胞治疗后融合基因再次转阴，末次随访时BCR-ABL1融合基因仍为阴性。1例患者移植后继续口服氟马替尼维持治疗。1例患者allo-HSCT后未满3个月，暂未启动氟马替尼维持治疗。3例患者因移植后分别出现皮肤、肝脏及肠道和肝脏GVHD，暂停TKI维持治疗。6例患者在治疗过程中均未发生CNSL。

**表2:** 6例新诊断Ph^+^急性淋巴细胞白血病患者氟马替尼联合allo-HSCT治疗结果

例号	氟马替尼起始剂量（mg/d）	服药前BCR-ABL1（％）	达MMR时间（d）	移植前疗效	服药至移植时间（d）	移植类型	移植后生存时间（d）	移植后氟马替尼应用	GVHD	有无合并CNSL或髓外浸润	末次BCR-ABL1（％）	存活状态
1	600	86.13	78	MMR	117	haplo-HSCT	521	18 d	无	无	0	LFS
2	600	114.75	12	MMR	103	haplo-HSCT	361	20 d	皮肤GVHD	无	0	LFS
3	600	109.18	12	CMR	89	Sib-HSCT	514	1个月	肝脏GVHD	无	0	LFS
4	600	128.57	59	MMR	104	haplo-HSCT	304	0	肠道、肝脏GVHD	无	0	LFS
5	600	105.73	18	CMR	171	haplo-HSCT	375	1年	无	无	0	LFS
6	600	100.79	50	CMR	161	haplo-HSCT	60	0	无	无	0	LFS

**注** MMR：主要分子学反应；CMR：完全分子学反应；haplo-HSCT：单倍体造血干细胞移植；Sib-HSCT：同胞全相合造血干细胞移植；GVHD：移植物抗宿主病；CNSL：中枢神经系统白血病；LFS：无白血病存活

三、不良反应与并发症

1. 诱导治疗期间：6例患者均出现Ⅳ级粒细胞减少；5例患者出现Ⅳ级贫血，1例患者出现Ⅲ级贫血；4例患者出现Ⅳ级血小板减少，1例患者出现Ⅲ级血小板减少，1例患者出现Ⅱ级血小板减少（表3）。6例患者中位粒细胞缺乏持续时间为9.5（4～12）d，PLT<20×10^9^/L的中位持续时间为7.5（0～15）d。1例出现谷丙转氨酶（ALT）和总胆红素（T-BIL）升高，1例出现肌酐（Cr-S）升高，2例出现一过性腹泻，1例出现皮疹，1例出现水肿与轻度四肢疼痛。6例患者中1例出现粒细胞缺乏伴发热。上述所有不良反应经对症治疗后均缓解。详见表3。

**表3:** 6例新诊断Ph^+^急性淋巴细胞白血病患者氟马替尼联合诱导化疗期间的不良反应发生情况

例号	粒细胞减少	贫血	血小板减少	肝功能异常	肾功能异常	腹泻	皮疹	水肿	四肢疼痛	心电图异常
1	Ⅳ级	Ⅳ级	Ⅳ级	−	−	+	−	−	−	−
2	Ⅳ级	Ⅳ级	Ⅳ级	−	Cr-S升高	−	−	−	−	−
3	Ⅳ级	Ⅳ级	Ⅳ级	ALT、T-BIL升高	−	−	−	+	+	−
4	Ⅳ级	Ⅳ级	Ⅳ级	−	−	−	−	−	−	−
5	Ⅳ级	Ⅳ级	Ⅲ级	−	−	−	+	−	−	−
6	Ⅳ级	Ⅲ级	Ⅱ级	−	−	+	−	−	−	−

**注** T-BIL：总胆红素；Cr-S：血清肌酐

2. 移植后单用氟马替尼治疗期间：4例患者（例1、2、3、5）移植后接受短暂氟马替尼治疗，例5出现持续Ⅱ级血红蛋白降低，其余3例均未出现血液学不良反应。1例患者出现轻度水肿和四肢疼痛，停药后恢复。1例患者出现轻度皮疹，其余2例患者未出现非血液学不良反应。详见表4。

**表4:** 4例新诊断Ph^+^急性淋巴细胞白血病患者移植后单用氟马替尼治疗期间的不良反应发生情况

例号	粒细胞减少	贫血	血小板减少	肝功能异常	肾功能异常	腹泻	皮疹	水肿	四肢疼痛	心电图异常
1	−	−	−	−	−	−	−	−	−	−
2	−	−	−	−	−	−	−	−	−	−
3	−	−	−	−	−	−	−	+	+	−
5	−	Ⅱ级	−	−	−	−	+	−	−	−

四、生存情况

所有患者接受氟马替尼治疗后中位随访时间为17（7～21）个月，移植后中位随访时间为12（2～17）个月，无失访病例。至随访终止，6例患者均处于无白血病存活状态。随访期间1例患者出现疾病进展（BCR-ABL1融合基因由阴转阳），经治疗后BCR-ABL1融合基因再次转阴。

## 讨论

TKI在Ph^+^ ALL治疗中已取得巨大成功。与传统单纯化疗相比，TKI在提高CR率的同时，显著降低了复发率，为患者赢得了序贯移植的机会[11]。Shen等[12]的一项Ⅲ期随机临床研究对一代TKI伊马替尼和二代TKI达沙替尼联合化疗治疗儿童Ph^+^ ALL的疗效进行了比较，结果显示达沙替尼组4年无事件生存（EFS）率、OS率、累积复发率均优于伊马替尼组（71％对48.9％、88.4％对69.2％、19.8％对34.4％）。一项Ⅱ期多中心临床试验结果显示，二代TKI达沙替尼联合化疗序贯allo-HSCT 3年OS率为75％[13]，优于日本学者报道[7]的一代TKI伊马替尼联合化疗序贯allo-HSCT（3年OS率为65％）。国内杨飞等[14]报道，TKI联合化疗并序贯移植治疗Ph^+^ ALL患者时，二代TKI组2年EFS率、OS率均优于一代TKI（84.2％对46.3％、94.7％对62.0％）。

氟马替尼是我国自主研制的一种新型TKI，在分子结构上，用吡啶环取代苯环，并导入三氟甲基，提高了氟马替尼与BCR-ABL1激酶的结合力，增强结合的稳定性，且氟马替尼比尼洛替尼具有更强的抗BCR-ABL1激酶的能力[15]–[16]。体外研究显示，氟马替尼对ABL激酶ATP结合区的突变（V299L、F317L和F317I）也有较强的抑制作用[16]。一项开放、随机、多中心Ⅲ期临床试验研究对比氟马替尼和伊马替尼治疗慢性髓性白血病慢性期（CML-CP）患者的安全性和疗效，结果显示氟马替尼组可获得更高、更快、更深的治疗反应且不良事件发生率更低[17]。米瑞华等用氟马替尼治疗Ph^+^ ALL（包括7例初诊和2例复发/难治Ph^+^ ALL），获得了较好的疗效。

本组6例初诊Ph^+^ ALL患者采用氟马替尼联合诱导化疗一线治疗28 d均获得形态学CR，治疗3个月均获得MMR（其中3例为CMR），提示该药在Ph^+^ ALL治疗领域可能具有较好的应用前景。既往研究显示，allo-HSCT前获得MMR的患者具有较好的移植后OS和LFS[18]。本组6例患者均在CR1期接受allo-HSCT，移植前未获得CMR的3例患者在移植后BCR-ABL1融合基因均转阴。1例患者在移植后4个月BCR-ABL1融合基因转阳，ABL激酶区出现T315I突变，予更换TKI，并先后予利妥昔单抗、CD19/22 CAR-T细胞治疗，融合基因再次转阴并维持至随访截止，该患者移植后已存活17个月。1例初诊时伴高白及IKZF1缺失的患者，采用氟马替尼联合诱导化疗序贯allo-HSCT，目前已存活12个月。1例患者在移植后重新启动氟马替尼维持治疗，安全性良好。6例患者在治疗期间均未出现Ⅲ级及以上非血液学毒性。

本研究结果初步显示了二代TKI氟马替尼联合诱导化疗并序贯allo-HSCT对初诊Ph^+^ ALL具有较好疗效且安全性良好。上述结论尚需前瞻性、多中心的临床研究加以证实。
